# Assembly of ordered DNA-curli fibril complexes during *Salmonella* biofilm formation correlates with strengths of the type I interferon and autoimmune responses

**DOI:** 10.1371/journal.ppat.1010742

**Published:** 2022-08-16

**Authors:** Lauren K. Nicastro, Jaime de Anda, Neha Jain, Kaitlyn C. M. Grando, Amanda L. Miller, Shingo Bessho, Stefania Gallucci, Gerard C. L. Wong, Çagla Tükel

**Affiliations:** 1 Center for Microbiology and Immunology, Lewis Katz School of Medicine, Temple University, Philadelphia, Pennsylvania, United States of America; 2 Department of Bioengineering, California Nano Systems Institute, University of California, Los Angeles, California, United States of America; 3 Department of Bioscience and Bioengineering, Indian Institute of Technology, Jodhpur, India; University of California Davis School of Medicine, UNITED STATES

## Abstract

Deposition of human amyloids is associated with complex human diseases such as Alzheimer’s and Parkinson’s. Amyloid proteins are also produced by bacteria. The bacterial amyloid curli, found in the extracellular matrix of both commensal and pathogenic enteric bacterial biofilms, forms complexes with extracellular DNA, and recognition of these complexes by the host immune system may initiate an autoimmune response. Here, we isolated early intermediate, intermediate, and mature curli fibrils that form throughout the biofilm development and investigated the structural and pathogenic properties of each. Early intermediate aggregates were smaller than intermediate and mature curli fibrils, and circular dichroism, tryptophan, and thioflavin T analyses confirmed the establishment of a beta-sheet secondary structure as the curli conformations matured. Intermediate and mature curli fibrils were more immune stimulatory than early intermediate fibrils *in vitro*. The intermediate curli was cytotoxic to macrophages independent of Toll-like receptor 2. Mature curli fibrils had the highest DNA content and induced the highest levels of *Isg15* expression and TNFα production in macrophages. In mice, mature curli fibrils induced the highest levels of anti-double-stranded DNA autoantibodies. The levels of autoantibodies were higher in autoimmune-prone NZBWxF/1 mice than wild-type C57BL/6 mice. Chronic exposure to all curli forms led to significant histopathological changes and synovial proliferation in the joints of autoimmune-prone mice; mature curli was the most detrimental. In conclusion, curli fibrils, generated during biofilm formation, cause pathogenic autoimmune responses that are stronger when curli complexes contain higher levels of DNA and in mice predisposed to autoimmunity.

## Author summary

Amyloid curli is an extracellular component of the biofilms of enteric bacteria like *Salmonella* and *E*. *coli*. During biofilm development, curli binds to extracellular DNA to form complexes that stimulate the immune system. Using complimentary techniques, we discovered that curli amyloids undergo changes in their physical and pathogenic properties throughout the development of the biofilm, polymerizing from early intermediate and intermediate to mature curli fibrils. While all three curli conformations activated TLR2, they bound different amounts of DNA that positively correlate with the complexity of the amyloids and their ability to induce type I IFNs and pro-inflammatory cytokines, and accelerate the onset of anti-dsDNA autoantibodies in autoimmune-prone mice. Synchrotron x-ray diffraction results suggest that DNA is organized by the beta sheet motif of curli monomers into a spatially periodic lattice that can amplify immune activation. Intermediate curli complexes were the only form that also showed cytotoxicity in macrophages independent of TLR2 and they induced higher levels of IL-1b, a cytokine involved in cell death. In mice prone to autoimmunity, the intermediate and mature curli/DNA induced also joint inflammation. Overall, the maturation of curli-DNA complexes through biofilm development stimulated distinct immune responses influenced by DNA content and promoted autoimmune features in mice.

## Introduction

Amyloid proteins adopt a conserved cross beta-sheet structure and form fibrils through a self-assembly process; the mature fibrils form beta-sheets that are oriented perpendicular to the axis of fibril growth [1]. There are more than 60 amyloidogenic proteins expressed in humans, but the normal physiological roles of most remain unclear. Fibrillar deposits of human amyloids are observed in various organs in patients with Alzheimer’s disease, Huntington’s disease, Parkinson’s disease, type II diabetes, and secondary amyloidosis [2–4]. Often amyloid fibrils or deposits are referred to as misfolded aggregates; however, amyloid fibrillization is a highly ordered process. Studies of amyloid β, found in senile plaques of Alzheimer’s disease patients, showed that in the earliest steps of amyloid fibrillization, amyloid β monomers associate to form oligomers. These oligomers then further polymerize to form protofibrils and then mature fibrils. The oligomeric forms of amyloid β are cytotoxic to immune cells through a mechanism that involves membrane permeation, whereas its fibrillar conformations interact with human innate immune receptors to induce inflammation [5–7].

Amyloid proteins are also produced by bacteria and are detected within the extracellular matrix of their biofilms [8]. Curli, the best-studied bacterial amyloid, is produced by enteric bacteria including *Escherichia coli* and *Salmonella enterica* serovar Typhimurium. Curli biogenesis requires the products of the *csg* gene cluster that act in the Type VIII secretion system [9, 10]. In a highly ordered process, CsgB, encoded by the *csgB* gene, nucleates the assembly of CsgA monomers, encoded by the *csgA* gene, into beta-sheet fibrils [9]. At high concentrations *in vitro*, purified CsgA monomers can self-assemble into fibrillar filaments in the absence of CsgB [11], but the molecular details of the assembly of CsgA into curli fibrils during the different stages of biofilm formation remain mostly unknown. Recently, our group demonstrated the presence of intermediate structures of curli that occur during biofilm formation by *S*. Typhimurium. Compared to mature curli fibril aggregates, the intermediate curli fibrils purified from the biofilm are smaller. Unlike mature curli fibrils, intermediate fibrils were cytotoxic to immune cells, similar to what is observed in experiments with human amyloid β oligomers [12]. Although fully formed mature curli fibrils are not cytotoxic to immune cells, they do elicit inflammation through their interactions with the heterocomplex of Toll-like receptors (TLR)-2 and TLR1 [13]. Thus, studies from eukaryotic and prokaryotic amyloid proteins suggest that different structures that form during amyloid fibrillization lead to different pathological outcomes during their interactions with the immune cells.

Curli fibrils bind extracellular DNA to form complexes during biofilm formation [14]. Curli/DNA complexes stimulate a stronger activation of dendritic cells than curli or DNA alone [15]. Mature curli/DNA complexes induce the upregulation of classical inflammatory cytokines such as IL-6, IL-12, and TNFα, induce the activation of the type I interferons and interferon-stimulated genes (ISGs), and also promote anti-double-stranded DNA (anti-dsDNA) and anti-nuclear autoantibody production, hallmarks of classical autoimmune responses [14, 15]. Recently, using a mouse model, we showed that curli-expressing bacteria initiate the onset of reactive arthritis [16], a human autoimmune disease that affects joints following gastrointestinal enteric infections by pathogens including *S*. Typhimurium, *Campylobacter jejuni*, and *Yersinia enterocolitica*. Recent work established that in mice infected with *S*. Typhimurium, systemic translocation of curli/DNA complexes is required for the generation of autoimmunity and joint inflammation [16]. Intriguingly, curli-expressing bacteria also participate in the pathogenesis of disease flares in patients with systemic lupus erythematosus (SLE). Persistent bacteriuria with uropathogenic *E*. *coli* and production of high levels of anti-curli/DNA antibodies were detected in SLE patients with higher markers of inflammation and increasing disease severity (flares)[17], suggesting that a systemic exposure to bacterial curli/DNA complexes can stimulate autoimmunity in SLE. These studies clearly provide a link between infections with curli-producing bacteria and autoimmune disease. Nevertheless, the inflammatory potential and the immunological roles of various conformations of curli, especially the cytotoxic intermediates, that are generated during biofilm formation remains unknown.

Here, using an interdisciplinary approach, we investigated how the various structural conformations of curli fibrils formed during biofilm formation interact with the immune system and we aimed to identify the conformations that elicit autoimmune responses. We determined that mature curli fibrils more efficiently induce autoantibody production than do early intermediates and intermediates, indicating that exposure to fully formed biofilm stimulates autoimmunity, and suggesting that DNA content influences this response. The increasingly polymerized structure of curli also induces higher production of pathogenic cytokines in autoimmunity, like type I IFNs and TNF**α** in macrophages. The levels of autoantibodies were higher in autoimmune-prone NZBWxF/1 mice than wild-type C57BL/6 mice and chronic exposure to all curli forms caused synovial proliferation in the joints of autoimmune-prone mice providing a link between genetic susceptibility, multiple aspects of autoimmunity and bacterial biofilms.

## Results

### Isolation of intermediates during curli fibril maturation

**Fig 1:**
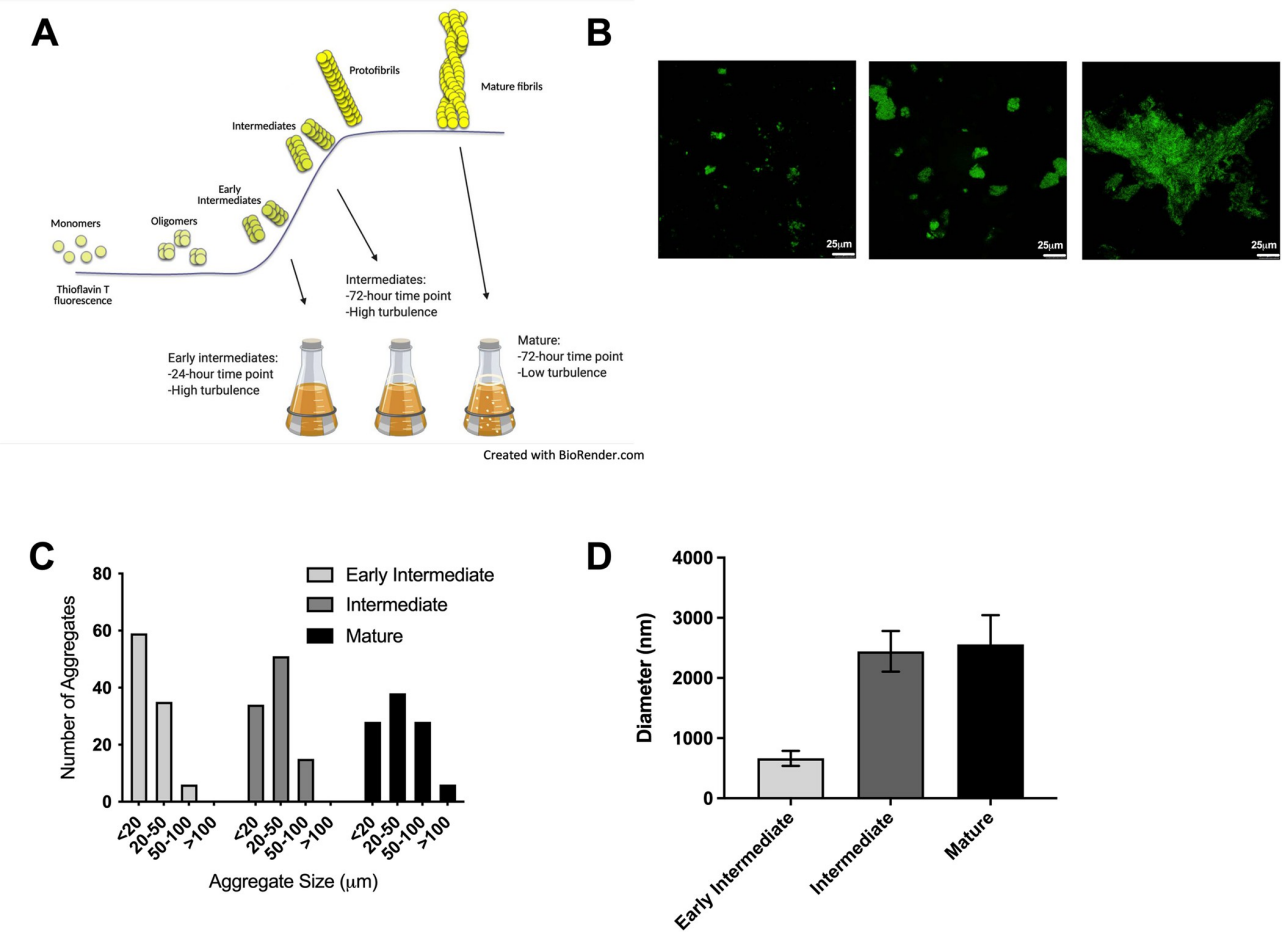
Characterization of sizes of curli-containing aggregates during the biofilm maturation. (A) Fibrillization curve showing the intensity of ThT stain, as a result of ThT incorporation, during maturation of the amyloid fibril from monomer to intermediate to mature fibrils. The bottom right of the image gives information about growth and collection conditions used to isolate the early intermediate, intermediate, and mature curli complexes. Aggregates are visible under conditions for collection of mature complexes. (B) Representative confocal microscopy images of samples of early intermediate (left), intermediate (middle), and mature (right) curli aggregates. Samples from 400 μg/mL stocks of each form were stained with 100 μM ThT and imaged at 60x magnification. Scale bars are 25 μm. (C) Number of aggregates at indicated sizes in samples of early intermediate, intermediate, and mature curli calculated from confocal microscopy images. One hundred aggregates were counted for each condition. (D) Diameters of aggregates in samples of early intermediate, intermediate, and mature curli determined by dynamic light scattering. The difference in diameters of intermediate and mature fibrils was not significant.

Amyloid monomers self-polymerize, first forming oligomers, then intermediates or protofibrils, and finally mature fibrils (Fig 1A). The unfolded and dynamic monomeric form of CsgA protein from *Salmonella* also undergoes a profound conformational change during amyloid formation. Due to nucleation with CsgB, CsgA polymerizes at a high rate under optimal conditions [18]. Extracellular DNA released during biofilm formation associates with the curli fibrils [14]; however, it remains unknown whether interactions with DNA influence fibril structure and/or immunogenicity.

Recently, we developed a novel technique to isolate and purify the intermediate forms of curli that arise during biofilm formation. Curli intermediates were enriched in smaller aggregates and lacked the larger aggregates observed in mature curli purified from an established biofilm [12]. We found that intermediate forms are cytotoxic to immune cells, whereas mature fibrils are not [12]. To understand the physical and immunological properties of curli as it forms into mature fibrils, we isolated curli at different stages of the biofilm formation. In addition to the previously isolated mature and intermediate fibrils, we were able to isolate a third conformation of curli, referred to here as early intermediate curli.

Briefly, in an effort to capture the earliest intermediates formed by CsgA, which we speculated to contain oligomeric units, we grew *S*. Typhimurium using high turbulence and a short incubation period of 24 hours (Fig 1A). The previously described curli intermediates were isolated at 72 hours under the same conditions (Fig 1A) [12]. Mature curli was isolated under low turbulence conditions, which allowed mature aggregates to form [12]. Samples were stained with the amyloid-specific fluorescent stain, thioflavin T (ThT), and imaged by confocal microscopy (Fig 1B). Due to the irregularity and asymmetry of the fibril aggregates, the lengths were measured along the longest axis of the aggregates. About 60% of the early intermediate aggregates had lengths of less than 20 μm; intermediate and mature samples were enriched in aggregates of 20 to 50 μm, and mature curli samples had aggregates larger than 100 μm (Fig 1B and 1C). We next performed dynamic light scattering experiments to obtain additional insight into the sizes of aggregates in the curli preparations. Large aggregates were removed using a 0.02-μm filter in all samples. In the early intermediate samples, the average diameter of aggregates was about 600 nm, whereas in intermediate and mature samples, the diameters were drastically larger at about 2500 nm (Fig 1D). After the removal of largest aggregates, we did not observe a significant difference in the diameter of intermediates and matured fibrils. The differences between these samples could be in the conformations of the polypeptide chains. From these data, we concluded that our purification protocol captured a previously uncharacterized early intermediate curli conformation.

**Fig 2:**
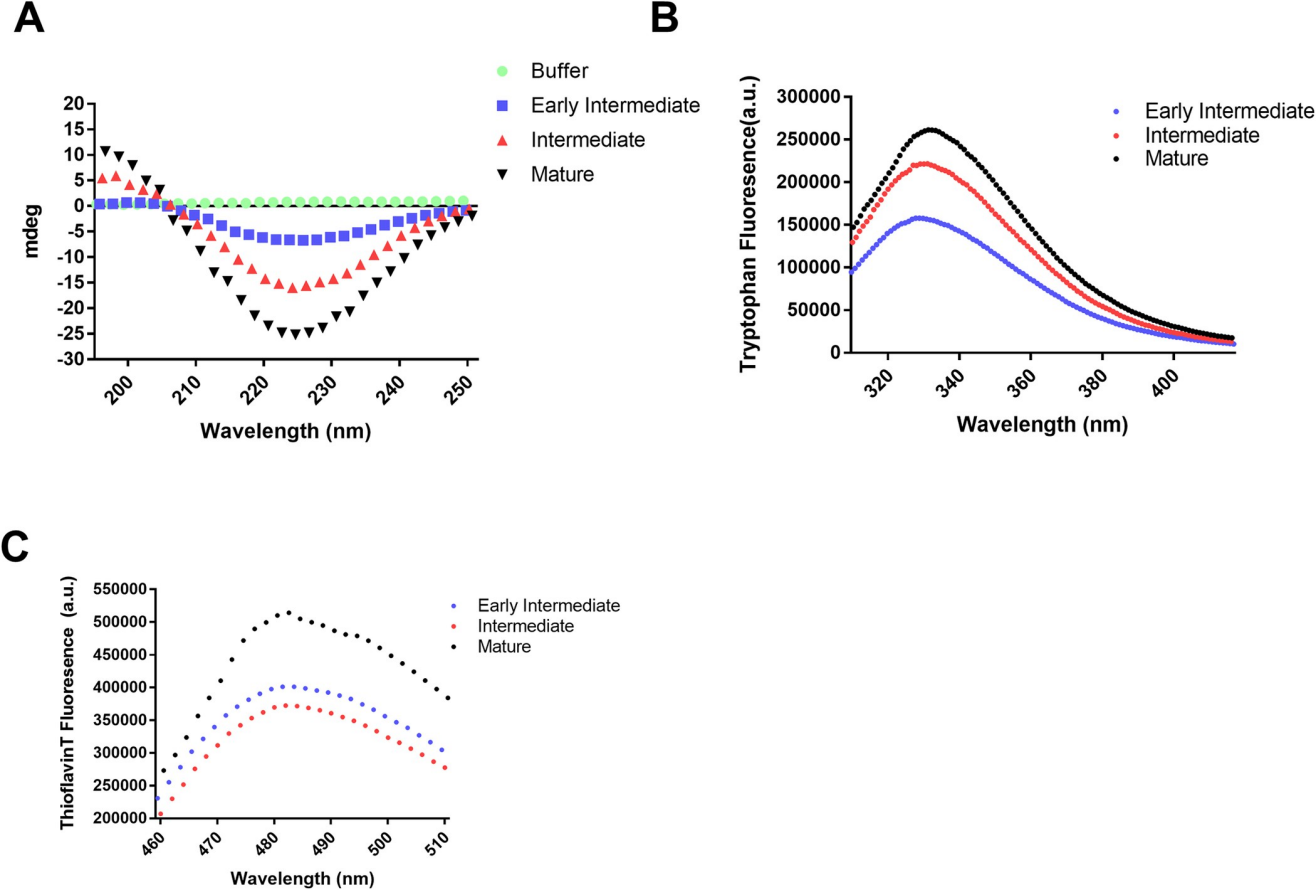
Characterization of curli structure over the course of fibril maturation. (A) UV circular dichroism spectra of mature (black triangles), intermediate (red triangles), and early intermediate curve (blue squares). The curve for buffer (green circles) is also shown. (B) Emission spectra of tryptophan in mature (black triangles), intermediate (red triangles), and early intermediate curve (blue squares) preparations. (C) ThT fluorescence in mature (black triangles), intermediate (red triangles), and early intermediate curve (blue squares) preparations.

We also investigated secondary structures of curli preparations using circular dichroism (CD) spectroscopy. In the CD spectrum of early intermediate samples, we observed a slight minima at about 225 nm; this negative peak was considerably more pronounced in intermediate and mature curli samples (Fig 2A). The signals at 225 nm and at 195 nm were more intense in mature curli samples than in intermediate samples (Fig 2A). These peaks are indicative of beta sheet secondary structure [19].

The rearrangement of polypeptide chain as a function of fibril maturation was further probed by tryptophan fluorescence. Tryptophan fluorescence is sensitive to the environment and is quenched in aqueous media but not in non-polar environments [20]. *Salmonella* CsgA has a single tryptophan located in the central region of the polypeptide chain (S1 Fig). This tryptophan can be used as a probe to monitor changes in protein conformation as it undergoes aggregation. The intensity of emission due to tryptophan was lowest in the early intermediate sample and highest in the mature curli sample (Fig 2B). This is consistent with greater aggregation in mature complexes than intermediate and early intermediate complexes.

To confirm the above results, we utilized ThT fluorescence as a probe to monitor aggregation and amyloid formation. ThT fluorescence is greatly enhanced upon its interaction with β-sheet-rich amyloid-like aggregates. Mature complexes showed significant enhancement in ThT fluorescence compared to intermediate and early intermediate samples indicating greater fibrillization (Fig 2C). Overall, these results confirmed an increase in the β-sheet content in the protein as it transitions from monomers and oligomers in the early intermediate stage to the mature amyloid-like fibril.

### DNA content increases during curli fibril maturation

**Fig 3:**
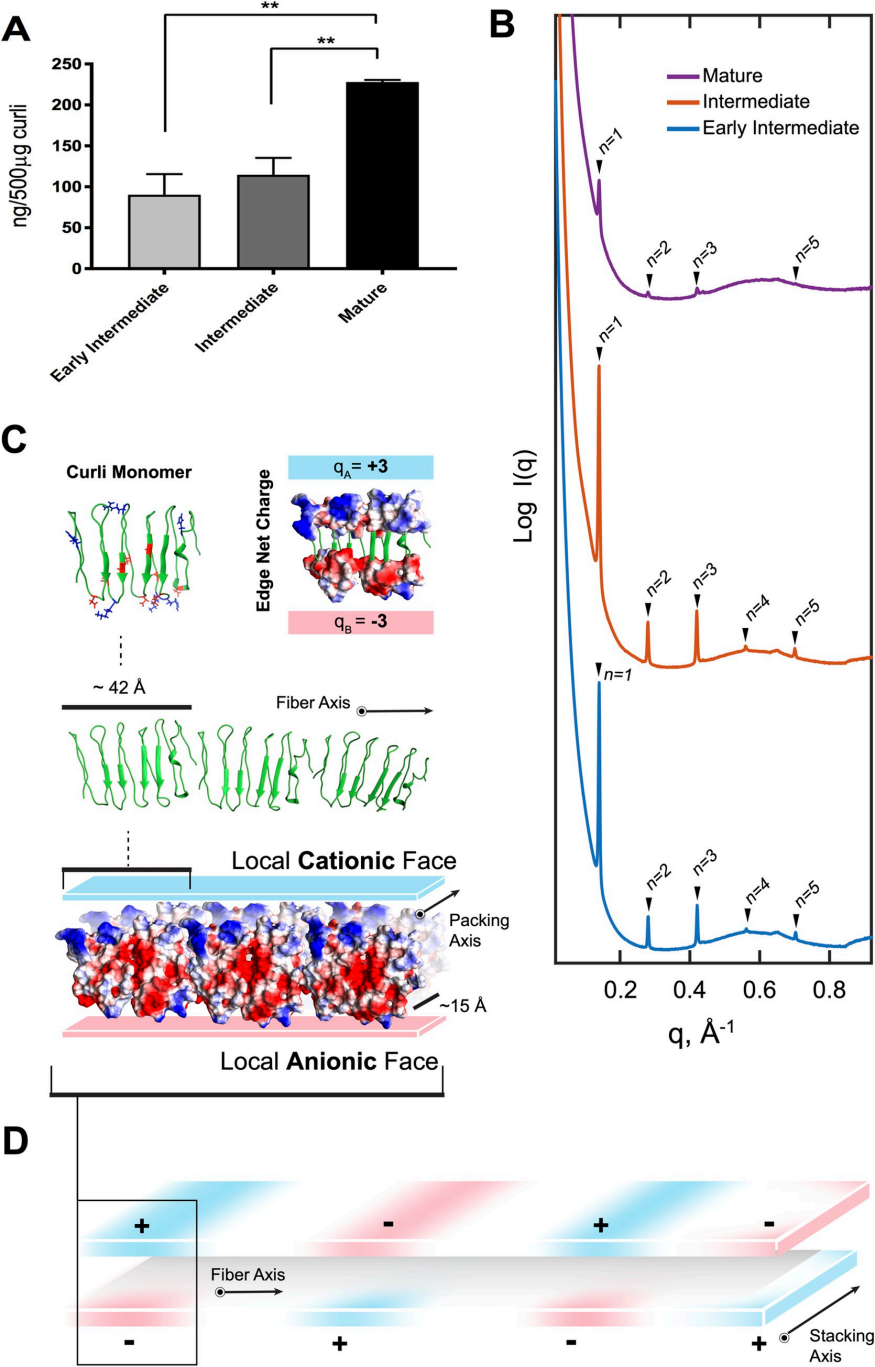
DNA associates with early intermediate, intermediate, and mature curli. (A) Amount of DNA in ng per 500 μg of curli preparation. Plotted are means (±SEM) of one representative experiment of three independent experiments. Statistical significance was calculated by ANOVA and Tukey post-hoc test. **, *P* < 0.01. (B) SAXS diffraction profiles for curli complexes. The mature curli preparation has four and early intermediate and intermediate have five Bragg peaks, and the scattering pattern matches a lamellar structure with characteristic spacing of 44.8 Å (q_1_ = 0.140 Å^-1^, q_n_ = n q_1_ where (n = 1,2,3,4,5)). Peaks are labeled with lamellar harmonics. To facilitate visualization, spectra were manually offset vertically by a multiplicative factor. (C) Molecular model of CsgA monomer indicating locations of charged amino acids at the beta-sheet turns (positive in blue and negative in red). The length of a monomer along the fibril axis is ~42 Å, creating a repeating charge along the fibril. Packing of fibrils gives rise to highly charged surfaces that repeat (into the page) every ~15 Å, which is the fibril thickness. (D) Model of alternating local cationic and anionic surfaces along the fibrils axis.

It has previously been shown that curli aggregates contain DNA [14]. We therefore determined the DNA content in the three curli preparations. As curli can be depolymerized only with high concentrations of formic acid which degrades the DNA [15], it is a challenge to accurately quantify DNA associated with the curli. We utilized phenol chloroform extraction to estimate the relative amounts of DNA within the curli preparations. Significantly more DNA was found in mature curli fibrils than in either early intermediate or intermediate preparations (Fig 3A).

In previous work, we performed structural studies of complexes formed when a synthetic CsgA peptide was fibrillized in the presence of DNA [14]. The partially ordered complex exhibited weak diffraction peaks that correspond to a periodicity of 41.6 Å, which we hypothesized was related to inter-DNA spacing [14]. Here, we used synchrotron-based small angle X-ray scattering (SAXS) to examine structures of DNA in curli preparations. The diffraction patterns from early intermediate, intermediate, and mature curli-DNA are similar and are characterized by five evenly-spaced peaks that correspond to characteristic diffraction peaks of a one-dimensional structure with a spatial periodicity of 44.8 Å (q_1_ = 0.140 Å^-1^, q_n_ = n q_1_ where (n = 1,2,3,4,5), Fig 3B). This observation suggests that an ordered, one-dimensional structural motif forms a common building block in all three samples. The sharpness of the diffraction features (FWHM = 3.8x10^-3^ Å^-1^) indicates large, ordered domain sizes. Given the high peptide-to-DNA concentration in the samples, the large molecular weight and polydispersity of DNA, we believe the observed structural motif stems from the ordering of curli fibril, which potentially provides a template for DNA ordering as described below.

Since a high-resolution structure of the CsgA monomer is not available, we modeled the CsgA monomer using RaptorX, a deep learning protein tertiary structure modeling tool [21, 22]. The predicted one-dimensional organization of monomers that comprise twisted beta sheets into a beta-helix fibril [23, 24] has a monomer periodicity of about 42 Å, which is consistent with previous models of CsgA (Fig 3C). Moreover, this predicted value of the periodicity is quite close to the one-dimensional spatial periodicity of 44.8 Å observed in our SAXS measurements.

It is interesting that curli and DNA form a complex, given that curli and DNA are both negatively charged. DNA, with a linear charge density of one negative charge per 1.7 Å, is one of the most anionic biological polymers. Such like-charge attraction is possible and has been observed before: when negatively charged proteins interact with DNA, multivalent cations such as Ca^2+^ are usually involved, since monovalent ions are known to screen and attenuate electrostatic repulsion but cannot generate attractions [25, 26]. Such multivalent cations are necessary for compensation of anionic charges on curli and DNA if neutral curli-DNA complexes are formed. What is more, the structure and dipolar charge distribution of curli β-sheet motifs deduced from SAXS experiments provide a natural template for ordering DNA into structured immunogenic complexes that activate TLR9, a Toll-like receptor activated by dsDNA. Each CsgA beta-sheet monomer has three cationic charges and three anionic charges at opposite edges of the beta sheet, therefore the beta sheet possesses an effective dipole moment perpendicular to the fibril direction. The local dipole moment slowly rotates with the twisting of the beta-sheets as monomer units are added to the fibril. We hypothesize that ordered, lateral packing of these one-dimensional CsgA beta-sheet fibrils creates localized, highly cationic ‘stripes’ which can interact with anionic DNA: each one-dimensional CsgA fiber is about 15 Å thick along this lateral packing direction (Fig 3C), so the effective local charge density along these stripes is approximately one positive charge per 5 Å. Electrostatic interaction between DNA and these cationic stripes results in a DNA sub-lattice with DNA oriented perpendicularly to the CsgA fibril axis, at an inter-DNA spacing of ~44.8 Å. This spacing is templated by the size of the monomer CsgA repeat, which is quite close to the predicted value of ~42Å). Interestingly, organized DNA ligands at this inter-DNA spacing have been previously shown to amplify TLR9 activation via multivalent DNA presentation [27, 28]. Since the transverse dipole moments are expected to slowly rotate along the CsgA fibril, we do not expect these local cationic domains to be large (Fig 3D).

### Intermediate, but not mature or early intermediate, curli is cytotoxic to immune cells

**Fig 4:**
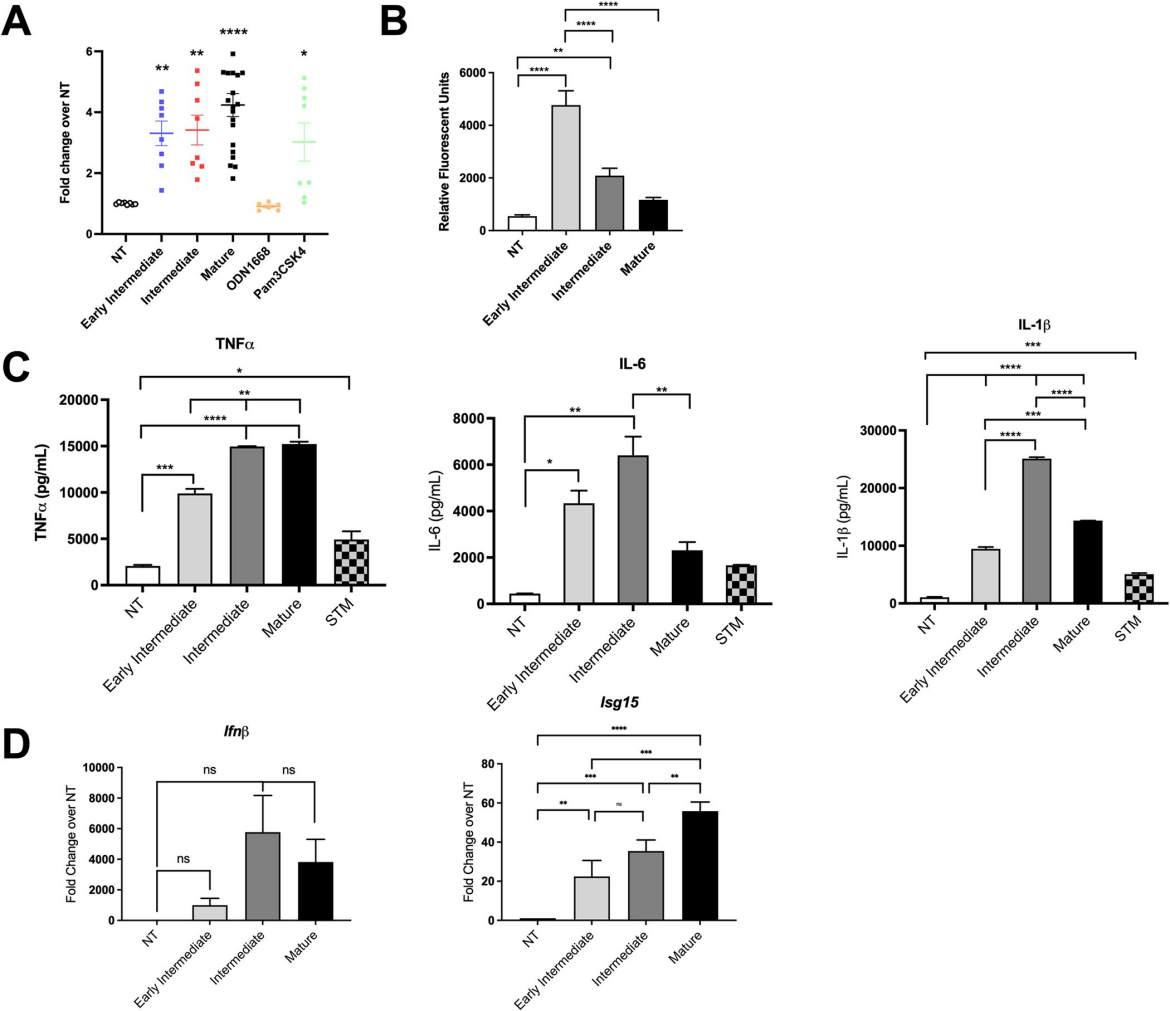
Curli preparations activate innate immune cells. (A) HEK293 Blue mTLR2 reporter cells (5x10^4^ cells/well) were treated with 10 μg/mL curli preparations, with CpG DNA (3 μg/mL) as a negative control, or with Pam_3_CSK_4_ (100 ng/mL) as a positive control. Absorbance at 620 nm was normalized to untreated (NT) control cells. Shown are data from four repeated experiments with whiskers showing SEM. Statistics were determined by ANOVA with Dunnett’s multiple comparisons post-test. *, *P* < 0.05; **, *P*<0.01; ****, *P* < 0.0001. (B) Wild-type BMDMs (5x10^5^ cells/well) were incubated with Congo Red-labeled curli preparations (10 μg/mL) and internalization was quantified by monitoring fluorescence relative to untreated (NT) control cells. Results are shown for four repeated experiments with SEM. Statistics were determined by ANOVA with Tukey’s post-hoc test. **, *P* < 0.01; ****, *P* < 0.0001. (C) Wild-type BMDMs (5x10^5^ cells/well) were stimulated with 10 μg of curli preparations or with *S*. Typhimurium (STM) grown in inflammasome inducing conditions at an MOI of 1:20 as a positive control or left untreated (NT) for 4 (IL-6 and TNFα) or 24 (IL-1β) hours. Production of TNFα (left), IL-6 (middle), and IL-1β (right) were quantified by ELISA in the supernatants. Means (±SEM) are shown. One representative experiment of three independent experiments is shown. (D) Wild-type BMDMs (5x10^5^ cells/well) were stimulated with 2.5 μg/mL of curli preparations or 3 μg/mL of CpG DNA or left untreated (NT). Expression of *Ifnβ* and *Isg15* was determined by qPCR.

The beta-sheet structure of curli activates the TLR2/TLR1 heterocomplex [13, 29]. To determine the capacity of curli conformations to activate TLR2, we utilized HEK293 Blue reporter cells that express TLR2. These cells report *NFκB* activation via the production of SEAP from a reporter gene; SEAP is detected using a chemiluminescence assay. *NFκB* activation was analyzed after the cells were treated with early intermediate, intermediate, and mature curli preparations for 24 hours. Treatments with curli preparations as well as Pam_3_CSK_4_, a TLR2 ligand, resulted in significant increases in secreted embryonic alkaline phosphatase (SEAP) expression compared to negative controls (Fig 4A). The highest NFκB activity was observed when the cells were treated with mature curli, suggesting that the maximal TLR2 engagement was achieved when curli fibrils reached their final amyloid structure.

TLR2 is involved in the phagocytosis of mature curli by murine macrophages [14]. To investigate the cellular immune responses to curli conformations that contain varying amounts of DNA, we first tested the internalization of the curli preparations using bone marrow-derived macrophages (BMDMs). BMDMs, isolated from wild-type C57BL/6 mice, were cultured with early intermediate, intermediate, and mature curli samples, previously stained with Congo Red, for 1 hour. The extracellular curli was removed by washing the cells with PBS. The internalization of the curli conformations was determined by measuring the Congo Red fluorescence from the lysed BMDMs. Fluorescence was significantly higher when the BMDMs were treated with the early intermediates compared to cells treated with intermediate or mature curli (Fig 4B), suggesting that the smaller aggregates were internalized significantly more readily by phagocytes. Next, we evaluated production of pro-inflammatory cytokines by BMDMs after treatment with curli preparations. Although the early intermediates were internalized at higher levels, they less effectively induced secretion of TNFα compared to intermediate or mature curli preparations (Fig 4C). The BMDMs treated with early intermediate and mature curli preparations showed similar production of IL-6 and IL-1β, which were significantly lower than levels secreted by cells treated with the intermediate curli preparation (Fig 4C). It is important to note that S. Typhimurium was used as a control to maximally activate the inflammasome and IL-1β production. Significantly higher levels of IL-1β were detected when the cells were stimulated with the curli intermediates compared to all treatments (Fig 4C)

We have previously shown that bacterial DNA associated with mature curli activates TLR9 and induces a type I IFN response [14]. We used quantitative real-time PCR (qPCR) to measure the expression of *Ifnβ*, which encodes a type I IFN, in curli-treated BMDMs to determine whether the DNA content in the curli complex alter the ability to stimulate a type I IFN response, possibly through the activation of TLR9. After 4-hour incubation with 2.5 μg/mL of curli preparations, *Ifnβ* expression was increased compared to untreated cells, with higher levels induced by mature and intermediate curli samples compared to early intermediate samples (Fig 4D). Although the early intermediates carry much less DNA compared to intermediate and mature curli, at this time point, we did not determine any significant changes in the expression of *Ifnβ*. However, significant changes in *Isg15* expression were determined which correlated with the DNA content of the curli conformations (Fig 4D).

**Fig 5:**
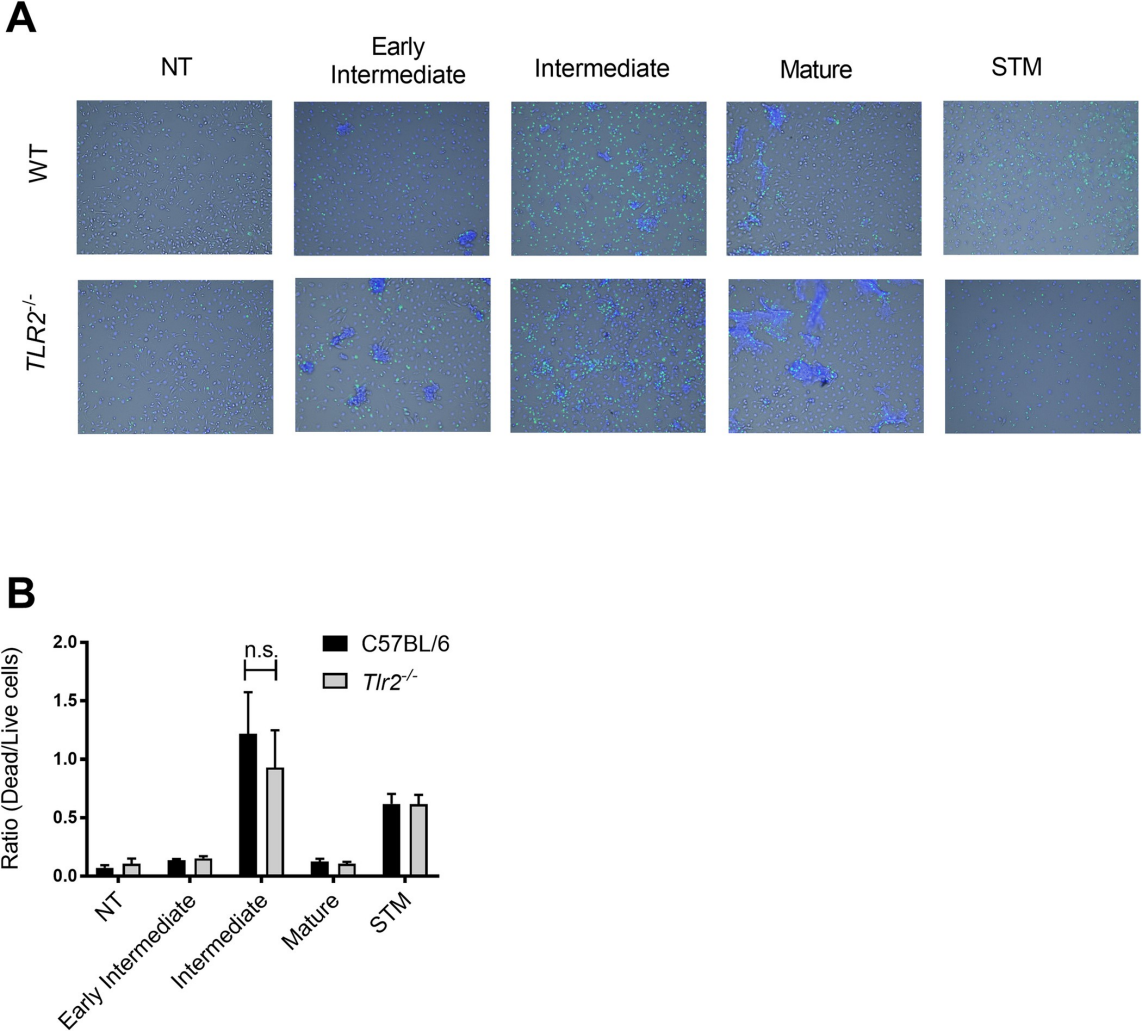
(A) Representative images of wild-type and *Tlr2*^-/-^ BMDMs (2x10^5^ cells/well) stimulated with 10 μg of curli preparations or *S*. Typhimurium grown under inflammasome inducing conditions or untreated (NT) for 24 hours. Cells were then stained with NucBlue (live nuclei) and NucGreen (dead nuclei) and imaged on the EVOS2 fluorescent microscope. (B) The ratios of dead to live cells obtained by analysis of multiple images from central 20% of each well per condition. Means of at least three independent experiments analyzed in duplicate are shown (±SEM).

In our previous study, we observed that the intermediates were more cytotoxic to macrophages than the mature curli fibrils [12]. As amyloid β oligomers have been shown to be more cytotoxic to immune cells than their more mature counterparts [5–7], we tested whether the early intermediates, which contain the smallest aggregates, are more cytotoxic than intermediate or mature curli conformations to BMDMs. BMDMs were treated with 10 μg of each curli preparation for 24 hours. Cells were stained with a live/dead stain and imaged using an EVOS2 fluorescent microscope. The ratio of dead to live cells was determined. Consistent with our previous observations, intermediate curli was significantly more cytotoxic than mature curli (Fig 5A and 5B). Intriguingly, the early intermediate preparation caused little to no cytotoxicity (Fig 5A and 5B). The cell death observed was comparable to that of the cells exposed to mature curli. Further, cytotoxicity of curli intermediates was TLR2 independent as the extent of death was similar in macrophage cultures from wild-type and *Tlr2*^*-/-*^ mice (Fig 5B). These data suggest that the cytotoxic effects of the intermediate conformations of curli do not result from the previously reported interaction with TLR2.

### Structure and DNA content of curli complexes dictate the autoimmune responses of mice

**Fig 6:**
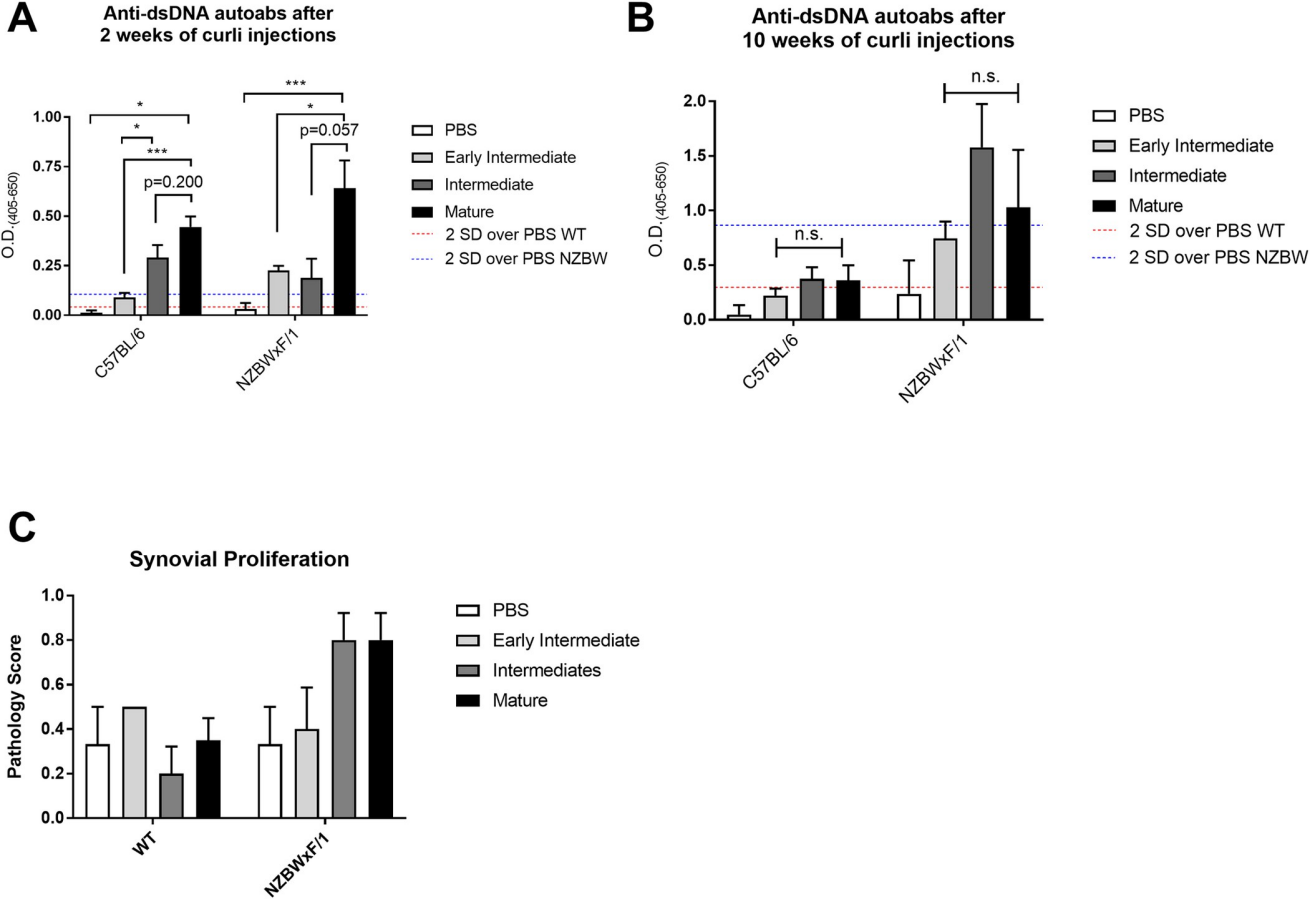
Structure and DNA content of curli complexes dictates the autoimmune response in mice. (A and B) Levels of anti-dsDNA autoantibodies in the sera of C57BL/6 and NZBWxF/1 mice treated with curli preparations twice weekly for A) 2 weeks and B) 10 weeks. For mice treated with curli preparations, n = 5; for PBS controls, n = 3. One representative experiment of three independent experiments is shown. (C) Joint pathology scores for C57BL/6 and NZBWxF/1 mice injected with 50 μg of early intermediate, intermediate, or mature curli preparations or PBS as a control twice weekly for 12 weeks. Scoring was based on histological parameters: 0, no changes; 1, slight thickening of synovial cell layer (< 3 layers of synoviocytes) accompanied by congestion and edema of the external membrane; 2, moderate thickening of synovial cell layer (3–5 layers of synoviocytes) accompanied by congestion and edema of the external membrane. Significance was determined by one-way ANOVA with Tukey post-hoc secondary test. *, *P* < 0.05; ***, *P* < 0.001; n.s. not significant.

We have previously established that the injection of mature curli fibrils elicits the generation of anti-dsDNA autoantibodies in mice in a TLR2- and TLR9-dependent manner [14]. As there are structural and DNA content differences in early intermediate, intermediate, and mature curli preparations that resulted in different abilities to stimulate the pro-inflammatory innate immune response and to induce immune cell death, we aimed to test whether these differences also affected the ability of curli to stimulate autoimmunity. First, we treated both C57BL/6 and autoimmune disease-prone NZBWxF/1 mice with 50 μg of curli preparations twice weekly for up to 10 weeks. Sera was collected every other week to analyze the autoantibody responses. After 2 weeks of injections, anti-dsDNA autoantibodies were detected in sera of both the wild-type and autoimmune-prone mice (Fig 6A). All three curli conformations elicited an autoantibody response compared to PBS-treated mice with the greatest response observed in mice treated with mature curli conformation, which carries the highest amount of DNA. Interestingly, after 10 weeks of continual treatment, the intermediate preparation induced an anti-dsDNA autoantibody response at levels similar to those of the mature complexes (Fig 6B). Whereas the wild-type mice produced similar levels of autoantibodies at 2-week and 10-week time points, the autoimmune-prone NZBWxF/1 mice produced higher levels of autoantibodies after 10 weeks of treatment with curli. This increase in autoantibodies was even more evident in NZBWxF/1 mice treated with the intermediate curli preparation, suggesting that the cytotoxic curli provides a stimulus that enhances the autoimmune process in mice genetically predisposed to autoimmune disease.

Recently, we showed that intraperitoneal injections of mature curli preparations induce joint inflammation in wild-type mice [15, 16]. Here, we investigated joint inflammation in wild-type and autoimmune-prone NZBWxF/1 mice treated with the three different curli preparations. Mice were injected intraperitoneally twice weekly with 50 μg of mature, intermediate, or early intermediate preparations of curli or PBS as a control. After 12 weeks of injections, knees were collected, fixed, decalcified, and paraffin embedded. Samples were scored for pathology for each treatment condition in a blinded fashion. The pathology scoring indicated that autoimmune-prone mice had greater synovial proliferation than wild-type mice, indicative of greater joint inflammation (Fig 6C). In NZBWxF/1 mice, treatment with both intermediate and mature curli induced the highest pathology scoring. It is important to note that wild type mice are resistant to arthritis and there were no significant changes observed among treatment groups. Overall, these data indicate that intermediate and mature curli fibrils induce autoimmune arthritis, especially in genetically predisposed mice, whereas the early intermediate curli preparation is much less inflammatory.

## Discussion

Bacteria form biofilms in environments such as the human gastrointestinal tract and at sites where medical devices have been implanted. In these tissues, the bacteria are exposed to the immune system and potentially antimicrobial agents. Biofilms allow bacteria to thrive in the presence of environmental hazards. The biofilm extracellular matrix protects the bacterial community by providing a strong and impenetrable shield [30]. The main protein component of the enteric biofilm extracellular matrix is the amyloid curli, which provides structural support and impenetrable matrix properties by forming a beta-sheet structure [31]. DNA as well as cellulose can bind to curli to provide additional support; however, the interactions between curli and these molecules are not well characterized [32, 33].

In a previous study, we were able to interfere with the polymerization of curli during biofilm formation and purified smaller curli aggregates that we termed intermediates. Curli intermediates were more cytotoxic to macrophages compared to mature fibrillar curli. However, the structural properties of these different curli conformations and their immunogenic capacity were not identified. In this study, we were able to isolate curli aggregates from an early stage of biofilm formation. By using a multidisciplinary approach, we investigated the DNA-binding capabilities and the structural properties of curli at early, intermediate, and late stages of biofilm development. We established that as curli matures, it forms the characteristic beta-sheet fibrillar structure, aggregates increase in size, and the fibrils incorporate more DNA. We observed that early intermediate, intermediate, and mature forms of curli all possess organization based on CsgA monomers ordered into one-dimensionally stacked, twisted beta-sheet motifs. (It is, however, possible that the quantitative amounts, spatial distributions, and organization of such motifs may be different for the various stages of fibril formation.) Importantly, ordered, lateral organization of these motifs can result in local cationic stripes that can bind and organize dsDNA via the entropy gain of counterion release [25, 26], resulting in an inter-DNA spacing that locally follows the periodicity of the CsgA monomer, a periodicity that falls in the optimum range for TLR9 immune activation via multivalent DNA presentation.

Curli and amyloid β have similar kinetics of fibrillization as well as functional and structural similarities [34]. The oligomeric conformations that form during the initial steps of polymerization of amyloid β are cytotoxic to immune cells, however, the early intermediate conformations of curli did not show this same increased cytotoxicity. The most cytotoxic conformation of curli was the intermediate form. Interestingly, the TLR2 receptor, which recognizes the beta-sheet structure of both amyloids [35, 36], was not involved in the cytotoxicity of the curli preparations, suggesting that alternative cytosolic cell death receptors such as inflammasomes or direct interactions with membranes mediate cell death. Consistent with this idea, earlier reports have shown that both curli and amyloid β activate the NLRP3 inflammasome. Although no cell death was observed upon activation of NLRP3 with curli or with amyloid β, these studies used the mature fibrillar forms of both amyloids [29, 37]. Whether early or intermediate forms of curli activate the NLRP3 inflammasome should be investigated.

It is important to note that biofilms observed *in vivo* have smaller aggregates than the large robust biofilms studied *in vitro*. It is possible that the cytotoxic curli intermediates kill immune cells and the DNA released from these cells is used by the bacteria to increase biofilm mass. Pathogenic bacteria such as *Haemophilus influenzae* and *Pseudomonas aeruginosa* incorporate neutrophil extracellular traps, which are sources of extracellular DNA, into their biofilms [38, 39]. However, it is not known whether the bacteria purposefully activate the neutrophils to induce production of neutrophil extracellular traps or if it is a coincidental activation.

Recently, it was reported that curli is expressed in the intestinal tract during enteric infections causing pathogenic effects [16]. During *Salmonella* infection, production of curli is associated with an increase in anti-dsDNA autoantibodies and joint inflammation in infected mice. Additionally, colonization of mice with *E*. *coli* cells that produce curli is associated with increased alpha synuclein deposition in the brain, possibly through the direct interactions of curli with the enteric vagus nerve [40, 41]. Our results indicate that different conformations of curli have different effects on the immune system. The anti-dsDNA autoantibody response was dependent on the DNA content of curli eliciting higher levels of anti-dsDNA autoantibodies at the early 2-week time point when treated with the mature preparation than did early intermediate or intermediate preparations. Treatment with early intermediate and intermediate curli preparations induced anti-dsDNA autoantibody production to levels similar to mature curli treatment at 10 weeks, suggesting that the production of antibodies by these different curli complexes work via different mechanisms. We attribute the high levels of anti-dsDNA autoantibody production induced by intermediate preparations of curli at the 10-week time point to the cytotoxicity associated with the fibrils, as the death of cells caused by this cytotoxic effect may provide a pool of nuclear material against which the autoantibodies can be formed. We suspect that chronic injections of curli lead to immune tolerance breakdown and a loss of autoantibody differences seen at earlier time points.

The observation that the repeated injections of curli over an extended time induces autoimmune responses in both healthy and genetically pre-disposed mice, but only in autoimmune prone mice they evolve in strength and severity, with increased levels of autoantibodies and development of signs like arthritis, supports the idea that biofilms carrying amyloid/DNA complexes or other protein/DNA complexes stimulate autoimmune responses by triggering specific immunity. Consistent with this hypothesis, infections with pathogenic bacteria such as *Borrelia burgdorferi*, *Mycobacterium tuberculosis* and *Streptococci* that produce curli-like amyloid proteins can lead to autoimmune sequelae [42–44]. Finally, these observations may not be specific to only bacterial amyloids. In *Clostridioides difficile* infections associated with colitis and intestinal inflammation, the pathogen’s exotoxin, toxin A (TcdA), organizes bacterial DNA into an optimal inter-DNA spacing to activate TLR9 [45]. In another example, the antimicrobial peptide (AMP) LL-37, which is essential for normal immune function and protection against lethal infections [46], has structural similarities to amyloids and forms insoluble complexes with DNA to activate TLR9 and promote type I IFN production [14, 47–50]. Chronic exposure to LL-37/DNA complexes leads to autoimmunity in SLE patients similar to described for curli/DNA complexes [15, 51].

One of the most common autoimmune sequelae observed following infections with Gram-positive and Gram-negative bacteria is post-infectious arthritis. After infections with invasive enteric bacteria such as *S*. Typhimurium resolve, 5–10% of the patients develop an inflammatory form of arthritis termed reactive arthritis (ReA). ReA is strongly associated with the HLA-B27 allele, indicating an important genetic susceptibility to this autoimmune disease [52]. The results that autoimmune prone mice progressively increase their autoantibodies levels with time, while the WT mice remain at initial levels, and have higher levels of joint inflammation than wild-type mice upon injections of curli indicate that genetic predisposition is important in the autoimmune responses stimulated by curli. Specifically, they suggest that, beside the traditional alterations in immune check points of B cell tolerance and in the antigen presentation of self-antigen by HLA alleles associated to higher risk to develop autoimmunity, such the HLA-B27 in Reactive Arthritis and DR3 and DRB1 in Systemic lupus erythematosus (SLE), genetic susceptibility may lead to abnormally strong reactions to bacterial curli.

Amyloid/DNA complexes are detected in biofilms of numerous bacteria, both commensal and pathogenic. Though the proteins themselves differ in primary amino acid sequence, amyloids self-assemble into a conserved beta-sheet structure and associate with DNA. Our demonstrations that immunological responses to intermediate and mature curli/DNA conformations generated throughout the biofilm development differ, and that genetically predisposed animals have exaggerated immune responses to curli/DNA complexes, provide novel insights into the link between bacterial infections and pathogenesis of autoimmune diseases, from the flares in Systemic lupus erythematosus to the induction of Reactive Arthritis.

## Materials and methods

### Ethics statement

All animal experiments were performed in a BSL2 facility with under protocols approved by AALAC-accredited Temple University Lewis Katz School of Medicine, Institutional Animal Care and Use Committee (IACUC #4561) in accordance with guidelines set forth by the USDA and PHS Policy on Humane Care and Use of Laboratory Animal Welfare (OLAW). The institution has an Animal Welfare Assurance on file with the NIH Office for the Protection of Research Risks (OPRR), Number A3594-01.

### Bacterial strains and growth conditions

The *S*. Typhimurium IR715 *msbB* mutant was previously described [53]. This strain was grown in Luria-Bertani broth (LB) supplemented with 100 μg/ml kanamycin at 37°C. A *bscE* cellulose mutant derived from the ATCC strain *S*. Typhimurium 14028, a gift from Dr. John Gunn (Nationwide Children’s Hospital, Columbus, OH), was used for SAXS experiments.

### Purification of curli

Curli aggregates were purified using a previously described protocol with slight modifications [12]. Briefly, an overnight culture of *S*. Typhimurium IR715 *msbB* was grown in LB with appropriate antibiotic selection with shaking (200 rpm) at 37°C. Overnight cultures were then diluted in yeast extract supplemented with casamino acids (YESCA) broth with 4% DMSO to enhance curli production [54]. Bacterial cultures were grown in either 150 ml of liquid YESCA medium containing 4% DMSO in a 250-ml flask or in 500 ml liquid YESCA medium in a 1-liter flask. These cultures were grown at 26°C for 72 h with shaking (200 rpm). Bacterial pellets were collected by centrifugation, resuspended in 10 mM Tris-HCl at pH 8.0, and treated with 0.1 mg/ml RNase A from bovine pancreas (Sigma, R5502), 0.1 mg/ml DNase I (Sigma, DN25), and 1 mM MgCl_2_ for 20 min at 37°C. Bacterial cells were then lysed by sonication (30% amplification for 30 s twice). Next, lysozyme was added (1 mg/ml; Sigma, L6876), and samples were incubated at 37°C. After 40 min, 1% SDS was added, and the samples were incubated for 20 min at 37°C with shaking (200 rpm). After this incubation, curli were pelleted by centrifugation (10,000 rpm in a J2-HS Beckman centrifuge with rotor JA-14 for 10 min at 4°C) and then resuspended in 10 ml Tris-HCl (pH 8.0) and boiled for 10 min. A second round of enzyme digestion was then performed as described above. The curli aggregates were then pelleted, washed in Tris-HCl at pH 8.0, and resuspended in 2× SDS-PAGE buffer and boiled for 10 min. The samples were then electrophoresed on a 12% separating/3 to 5% stacking gel run for 5 h at 20 mA (or overnight at 100 V). Fibrillar aggregates are too large to pass into the gel and therefore remain within the well of the gel and can be collected. Once collected, the curli aggregates were washed three times with sterile water and then extracted twice with 95% ethanol. Curli preparations were then resuspended in sterile water. Concentrations of curli aggregates were determined using the bicinchoninic acid (BCA) assay according to the manufacturer’s instructions (Novagen, 71285–3). Curli protein preparations were adjusted to be 1mg/ml and the protein amount was confirmed by Western blot.

### Confocal laser scanning microscopy

For confocal images, curli preparations were stained with a 1:1 v/v ratio of ThT to curli (400 μg/mL), and 5 μL was spotted onto a microscopy slide. Spots were allowed to dry completely and then were analyzed on the Leica SP5 microscope with a TCS confocal system. For enumeration of aggregates by size, the LAS AF confocal system was used to draw scale bars on aggregates from multiple fields, and aggregate sizes were recorded: 100 aggregates per condition.

### Dynamic light scattering

The size of the aggregating species was monitored using a Malvern Zetasizer Nano-ZS90 instrument. Samples were excited using a He−Ne laser (632 nm). The buffer and protein were filtered through 0.02-μm filters before experiments. For measurements, the sample concentration was ~1 mg/ml.

### Ultraviolet circular dichroism spectroscopy

The UV CD measurements were taken on a Chirascan CD spectrometer (Applied PhotoPhysics) at room temperature (~25°C). The spectra were recorded in quartz cuvettes with 1-mm pathlength with a scan range from 195 nm to 260 nm and a scan rate of 2 nm/s. All the scans were taken at ~1 mg/ml concentration. For each curli preparation, three spectra were averaged and subtracted from blank (buffer) using Chirascan ProData viewer software provided with the instrument.

### Tryptophan fluorescence assay

We performed steady-state fluorescence measurements to probe CsgA conformations in curli preparations. A FluoroMax-4 (Horiba) fluorescence spectrophotometer was used to record spectra. The following parameters were set for the measurements: lex = 295 nm, emission scan range = 310–420 nm, excitation bandpass = 1.5 nm, and emission bandpass = 2 nm. The integration time was set to 2 s with a 1-nm increment. The final concentration of samples was ~1 mg/ml. Cuvettes had 2-mm pathlength.

### Thioflavin T fluorescence assay

The extent of amyloid formation in curli preparations was monitored using a ThT assay. The concentrations of protein and ThT were ~1 mg/ml and 10 μM, respectively. The experiments were performed on a FluoroMax-4 (Horiba) fluorescence spectrophotometer with the following parameters: lex = 440 nm, lem = 500 nm.

### DNA extraction

DNA was extracted using a previously described method [12]. Briefly, 500 μg of curli preparation based on the BCA assay in sterile water was centrifuged at 10,600 rcf (Eppendorf 5804R) for 3 min to pellet. The pellets were then resuspended in 550 μL of TE buffer, 30 μL of 10% sodium dodecyl sulfate, and 20 μL of 20 mg/mL proteinase K and mixed gently. This mixture was incubated at 37°C for 1 h. After incubation, 100 μL of 5 M NaCl and 80 μL of cetrimonium bromide were added. After incubation at 37°C for 10 min, 300–400 μL of phenol-chloroform-isoamyl alcohol was added. After mixing, the samples were centrifuged at 15,200 rcf (Eppendorf 5804R) for 5 min at 4°C. The supernatant was transferred to a clean 1.5-mL tube, and 700 μL of chloroform was added. The solution was mixed and centrifuged. The supernatant was transferred to a clean tube, equal volumes of isopropanol were added, followed by gentle mixing. The mixture was then incubated at -20°C for at least 30 min to precipitate the DNA. The DNA was pelleted at 15,200 rcf (Eppendorf 5804R) for 5 min at 4°C. The pellet was washed with 70% ethanol and centrifuged at 6000 rcf (Eppendorf 5804R) for 5 min. The supernatant was removed and pellet was resuspended in 30 μL of TE buffer. The final DNA concentration was determined from absorbance measured using a NanoDrop2000 spectrophotometer.

### Small-angle X-ray scattering analysis

Curli preparations were isolated from biofilm pellicles of liquid cultures of the *bscE* cellulose mutant of *S*. Typhimurium as described above. SAXS analysis was performed as previously described [14]. Briefly, complexes were pelleted in 1.5-mL tubes for 20 min at 10,000 rpm in a microfuge. Supernatants were discarded except for the last 50 μL. The complexes were resuspended in the remaining supernatant. These samples were then loaded into and sealed in quartz capillaries (Mark-tubes) and stored at 4°C until measurement. SAXS experiments were performed at the Stanford Synchrotron Radiation Lightsource (SSRL, Beamline 4–2) using 9-keV monochromatic X-rays. The scattered radiation was measured using a Rayonix MX-225-HE detector (pixel size 73.2 μm). Two-dimensional powder diffraction patterns were integrated using the Nika 1.74 [55] package for Igor Pro 6.37 and FIT2D [56]. SAXS data were analyzed by plotting integrated scattering intensity against the momentum transfer *q* using MATLAB. The characteristic spacing *d* was obtained from the first peak position *q*_*(1)*_ by the formula *a* = 2π/*q*_*(1)*_.

### HEK293 Blue NFκB Reporter cell assay

HEK293 Blue mTLR2 SEAP reporter cells (Invivogen, hkb-mtlr2) were cultured according to the manufacturer’s protocol. Curli preparations were added to wells of a 96-well plate at a concentration of 10 μg/mL. Volumes were adjusted to 20 μL with PBS. Reporter cells were added to wells at 5x10^5^ cells/well in HEK Detection media (Invivogen, hb-det2) to a final volume of 200 μL. Treated cells were incubated at 37°C with 5% CO_2_ for 24 h. The absorbance at 620 nm was measured to determine NFκB activation. CpG ODN1668 (Invivogen, tlrl-1668), referred to here as CpG DNA, is a mTLR9 ligand used as a negative control. Pam_3_CSK_4_ (Invivogen, tlrl-pms) is a mTLR2 ligand used as a positive control.

### Analysis of internalization of Congo Red-labeled curli

To quantify the internalization of curli-DNA complexes, wild-type BMDMs were seeded in a 48-well polystyrene plate (Costar, 3524) at 1x10^6^ cells per well. Cells were stimulated with Congo Red-labeled curli for 1 h. Cells were washed three times with sterile PBS to remove any extracellular labeled curli-DNA complexes and then were lysed with sterile PBS supplemented with 1% Triton-X. Lysates were transferred to a clear-bottom, black, 96-well microplate, and fluorescence of Congo Red was measured using a Flex Station (Molecular Devices) with excitation at 497 nm and emission at 614 nm. Results are reported as relative fluorescent units.

### Cytokine secretion and gene expression analyses using bone marrow-derived macrophages

BMDMs from C57BL/6 and *Tlr2*^-/-^ mice were differentiated as previously described [57]. Briefly, leg bones were flushed with RPMI, and a single cell suspension of the bone marrow was prepared in RPMI. The cell suspension was then centrifuged at 170 rcf (Sorvall TC6) for 10 min to pellet the marrow cells. These cells were then resuspended in RPMI supplemented with L929 conditioned media and antibiotic/antimycotic. On day 4, 5 mL of bone marrow differentiation media was added. On day 7, cells were seeded into 24-well plates at a density of 500,000 cells/well.

To investigate cytokine response, BMDMs were stimulated with 2.5 μg/mL of curli preparation or not treated. After 4 h, IL-6 and TNFα were quantified in the supernatant by ELISAs according to the manufacturer’s protocol (eBiosciences). After 24 h, IL-1β was quantified by ELISA (eBiosciences) according to the manufacturer’s protocol. To investigate type I IFN expression, *Ifnβ* mRNA was quantified at 4 h as described below. For cytotoxicity experiments, cells were stimulated with 10 μg of curli preparation for 24 h. Live/dead staining experiments were completed as described below. Cells treated with *S*. Typhimurium as a positive control were treated at time 0 and then treated with 5 μg/mL of gentamycin after 1 h to kill any bacteria that had not been taken up by the macrophages.

### RNA isolation and qPCR quantification

RNA was isolated from BMDMs using TriReagent according to the manufacturer’s instructions. Briefly, all surfaces were sprayed and cleaned with RNase Zap (Thermo Fisher, AM97890). An aliquot of 500 μL of TriReagent (Molecular Research Center, TR-118) was added to a monolayer of 5x10^5^ cells. The homogenate was stored at room temperature for 5 min. Using RNase-free, filter tips, the TriReagent was moved to an RNase-free Eppendorf tube. To the TriReagent homogenate, 100 μL of chloroform was added. After mixing, tubes were allowed to sit on the benchtop until phase separation occurred and were then centrifuged for 15 min at 4°C at 12000 rcf. The clear aqueous phase was carefully removed and added to 500 μL of isopropanol pre-chilled to -20°C. After shaking briefly, tubes were incubated at -20°C for 30 min and then centrifuged at 12000 rcf for 8 min at 4°C. The supernatant was removed, and the pellet was washed with 1 mL of 75% ethanol and then centrifuged at 7500 rcf for 5 min at 4°C. The ethanol was removed by aspiration, and pellets were allowed to air dry to allow. Dried pellets were resuspended in 30 μL of molecular-grade water.

RNA was then treated with DNase according to the manufacturer’s protocol (Ambion, AM1906). In short, 3 μL of 10x buffer with 1 μL of DNase I enzyme was added to each sample. Samples were incubated at 37°C for 30 min. Next, 3 μL of inactivation reagent was added to each sample, mixed, and incubated at room temperature for 5 min. Samples are then centrifuged at 10,000 rcf for 1 min. The RNA in the supernatant quantified by analysis of absorbance using a NanoDrop spectrophotometer.

The RNA was then reverse transcribed to cDNA using a TaqMan Reverse Transcription kit according to manufacturer’s protocol (Invitrogen, N8080234). In brief, 1 μg of RNA was dissolved in 19.25 μL molecular grade water. An aliquot of 30.75 μL master mix of MgCl_2_, 10X reverse transcription buffer, RNase inhibitor, reverse transcriptase enzyme, and random hexamers was added to each sample. A BioRad S100 Thermo Cycler was used to generate the cDNA (Step 1: 25°C for 10 min, Step 2: 48°C for 30 min, Step 3: 95°C for 5 min, Step 4: 4°C hold).

For qPCR, master mix for each sample included 0.75 μL of 100 μM forward primer and 0.75 μL of 100 μM reverse primer (Table 1), 12.5 μL Power Up Sybr Green master mix (ThermoFisher, A25742), and 6 μL molecular-grade water. In an 8-tube PCR strip, 20 μL of master mix and 5 μL of RNA were mixed. Aliquots of 10 μL were transferred to a MicroAmp qPCR plate in duplicate. qPCR was performed with an Applied Biosciences StepOne Plus Real Time PCR system. Transcript levels were determined using the ΔC_T_ approach.

**Table 1:** Primer sequences used for qPCR.

Gene Target	Direction	Sequence	Source
*Ifnβ*	Forward Reverse	5’ CAG CTC CAA GAA AGG ACG AAC 3’ 5’ GGC AGT GTA ACT CTT CTG CAT 3’	Harvard Primer Bank ID: 6754304a1 Harvard Primer Bank ID: 6754304a1
*GAPDH*	Forward Reverse	5’ CCA GGA AAT CAG CTT CAC AAA CT 3’ 5’ CCC ACT CCT CCA CCT TTG AC 3’	• [58] • [58]

### Live/Dead staining analysis

Cells were stained using the ReadyProbes cell viability imaging kit (Thermo Fisher, R37609) following the manufacturer’s instructions. Briefly, 2 drops of each color stain were added per 1 ml of medium, and stain was aliquoted into each well and incubated at room temperature for 15 min protected from light. Cells were then imaged on the EVOS FL Auto 2 microscope. Analysis of images to determine the percentage of dead cells to live cells was done using the HCS Studio software system.

### Animal experiments

To evaluate autoantibody production by mice in response to systemic exposure to curli preparations, female C57BL/6 (wild-type) and lupus-prone NZBWxF/1 mice were injected intraperitoneally with 50 μg of curli preparation in 100 μL of sterile PBS or with 100 μL of sterile PBS twice a week for 12 weeks. Tail bleeds were used to collect sera before first injection and once every 2 weeks throughout the experiment.

### Analysis of anti-dsDNA autoantibodies by ELISA

ELISAs were performed according to previously published protocol [59]. Briefly, a 96-well plate (Costar, 07-200-33) was coated with 0.01% poly-L-lysine (Sigma, P8920) in PBS and incubated for 1 h at room temperature. Plates were then washed three times with distilled water and dried. Plates were then coated with 2.5 μg/mL of calf thymus DNA (Invitrogen, 15633–019) in borate buffered saline (BBS) (17.5 g NaCl, 2.5 g H_3_BO_3_, 38.1 g sodium borate in 1 L H_2_O) and incubated at 4°C overnight. The next day, plates are washed three times with BBS and blocked with 200 μL/well of BBT (BBS with 3% bovine serum albumin and 1% Tween 20) for 2 h at room temperature with gentle rocking. After washing five times with BBS, serial dilutions of control serum and sample sera were incubated on the plate overnight at 4°C. Next, plates were washed three to five times with BBS, and biotinylated goat anti-mouse IgG (Jackson ImmunoRes, 115-065-071) was added. Samples were incubated at room temperature for 2 h with gentle rocking. Avidin-alkaline phosphate conjugate (Sigma, A7294) was added. After 2 h at room temperature, plates were washed five times with BBS, and then 4-nitrophenyl phosphate disodium salt hexahydrate (Sigma-Aldrich, N2765) was added to the plate at 1 mg/mL. Plates were incubated at 37°C protected from light for at least 3 h. Optical densities at 650 nm and 405 nm were determined using a Molecular Devices Microplate reader. Positive control sera were taken from B6.NZM Sle1/Sle2/Sle3 lupus-prone mice with high levels of autoantibodies and used at a dilution of 1:250 in BBT.

### Joint inflammation analysis

Murine knees were extracted and fixed in phosphate-buffered formalin. For decalcification, samples were incubated in formic acid for 3 days and then embedded in paraffin. Sections of 5 μm of the tissue were stained with hematoxylin and eosin. The fixed and stained sections were blinded and evaluated by an experienced veterinary pathologist according to the criteria previously described [60]. Images were taken at a magnification of 10x.

### Statistical analyses

Data were analyzed using Prism software (GraphPad). Student’s *t* test or one-way ANOVA were used when appropriate. Error was determined by standard error of the mean. *P* values of <0.05 were considered significant and were noted as such on figures.

## Supporting information

S1 Fig A single tryptophan in CsgA monomer. The amino acid structure of the CsgA monomer emphasizing the single tryptophan within the sequence used for the analysis of polymerization.
(JPEG)

## References

[1] Schnabel J. Protein folding: The dark side of proteins. Nature. 2010;464(7290):828–9. doi: 10.1038/464828a . 20376124

[2] Ross CA, Poirier MA. Protein aggregation and neurodegenerative disease. Nat Med. 2004;10 Suppl:S10–7. Epub 2004/07/24. doi: 10.1038/nm1066 [pii]. . 15272267

[3] Hull RL, Westermark GT, Westermark P, Kahn SE. Islet amyloid: a critical entity in the pathogenesis of type 2 diabetes. J Clin Endocrinol Metab. 2004;89(8):3629–43. doi: 10.1210/jc.2004-0405 . 15292279

[4] Cherny I, Rockah L, Levy-Nissenbaum O, Gophna U, Ron EZ, Gazit E. The formation of Escherichia coli curli amyloid fibrils is mediated by prion-like peptide repeats. J Mol Biol. 2005;352(2):245–52. doi: 10.1016/j.jmb.2005.07.028 . 16083908

[5] Xue WF, Hellewell AL, Hewitt EW, Radford SE. Fibril fragmentation in amyloid assembly and cytotoxicity: when size matters. Prion. 2010;4(1):20–5. doi: 10.4161/pri.4.1.11378 ; PubMed Central PMCID: PMC2850416. 20305394 PMC2850416

[6] Bucciantini M, Giannoni E, Chiti F, Baroni F, Formigli L, Zurdo J, et al. Inherent toxicity of aggregates implies a common mechanism for protein misfolding diseases. Nature. 2002;416(6880):507–11. Epub 2002/04/05. doi: 10.1038/416507a [pii]. . 11932737

[7] Balducci C, Beeg M, Stravalaci M, Bastone A, Sclip A, Biasini E, et al. Synthetic amyloid-beta oligomers impair long-term memory independently of cellular prion protein. Proc Natl Acad Sci U S A. 2010;107(5):2295–300. doi: 10.1073/pnas.0911829107 ; PubMed Central PMCID: PMC2836680. 20133875 PMC2836680

[8] Barnhart MM, Chapman MR. Curli biogenesis and function. Annu Rev Microbiol. 2006;60:131–47. doi: 10.1146/annurev.micro.60.080805.142106 ; PubMed Central PMCID: PMC2838481. 16704339 PMC2838481

[9] Chapman MR, Robinson LS, Pinkner JS, Roth R, Heuser J, Hammar M, et al. Role of Escherichia coli curli operons in directing amyloid fiber formation. Science. 2002;295(5556):851–5. Epub 2002/02/02. doi: 10.1126/science.1067484 ; PubMed Central PMCID: PMC2838482. 11823641 PMC2838482

[10] Desvaux M, Hebraud M, Talon R, Henderson IR. Secretion and subcellular localizations of bacterial proteins: a semantic awareness issue. Trends Microbiol. 2009;17(4):139–45. doi: 10.1016/j.tim.2009.01.004 . 19299134

[11] Wang X, Smith DR, Jones JW, Chapman MR. In vitro polymerization of a functional Escherichia coli amyloid protein. J Biol Chem. 2007;282(6):3713–9. Epub 2006/12/14. doi: 10.1074/jbc.M609228200 ; PubMed Central PMCID: PMC2838475. 17164238 PMC2838475

[12] Nicastro LK, Tursi SA, Le LS, Miller AL, Efimov A, Buttaro B, et al. Cytotoxic Curli Intermediates Form during Salmonella Biofilm Development. J Bacteriol. 2019;201(18). doi: 10.1128/JB.00095-19 ; PubMed Central PMCID: PMC6707925. 31182496 PMC6707925

[13] Tukel C, Nishimori JH, Wilson RP, Winter MG, Keestra AM, van Putten JP, et al. Toll-like receptors 1 and 2 cooperatively mediate immune responses to curli, a common amyloid from enterobacterial biofilms. Cell Microbiol. 2010;12(10):1495–505. doi: 10.1111/j.1462-5822.2010.01485.x ; PubMed Central PMCID: PMC3869100. 20497180 PMC3869100

[14] Tursi SA, Lee EY, Medeiros NJ, Lee MH, Nicastro LK, Buttaro B, et al. Bacterial amyloid curli acts as a carrier for DNA to elicit an autoimmune response via TLR2 and TLR9. PLoS Pathog. 2017;13(4):e1006315. doi: 10.1371/journal.ppat.1006315 ; PubMed Central PMCID: PMC5406031. 28410407 PMC5406031

[15] Gallo PM, Rapsinski GJ, Wilson RP, Oppong GO, Sriram U, Goulian M, et al. Amyloid-DNA Composites of Bacterial Biofilms Stimulate Autoimmunity. Immunity. 2015;42(6):1171–84. Epub 2015/06/18. doi: 10.1016/j.immuni.2015.06.002 ; PubMed Central PMCID: PMC4500125. 26084027 PMC4500125

[16] Miller AL, Pasternak JA, Medeiros NJ, Nicastro LK, Tursi SA, Hansen EG, et al. In vivo synthesis of bacterial amyloid curli contributes to joint inflammation during S. Typhimurium infection. PLoS Pathog. 2020;16(7):e1008591. Epub 2020/07/10. doi: 10.1371/journal.ppat.1008591 ; PubMed Central PMCID: PMC7347093. 32645118 PMC7347093

[17] Pachucki RJ, Corradetti C, Kohler L, Ghadiali J, Gallo PM, Nicastro L, et al. Persistent Bacteriuria and Antibodies Recognizing Curli/eDNA Complexes From Escherichia coli Are Linked to Flares in Systemic Lupus Erythematosus. Arthritis Rheumatol. 2020;72(11):1872–81. Epub 2020/08/26. doi: 10.1002/art.41400 ; PubMed Central PMCID: PMC7722165. 32840064 PMC7722165

[18] Hammer ND, McGuffie BA, Zhou Y, Badtke MP, Reinke AA, Brannstrom K, et al. The C-terminal repeating units of CsgB direct bacterial functional amyloid nucleation. J Mol Biol. 2012;422(3):376–89. doi: 10.1016/j.jmb.2012.05.043 ; PubMed Central PMCID: PMC3423549. 22684146 PMC3423549

[19] Tukel C, Wilson RP, Nishimori JH, Pezeshki M, Chromy BA, Baumler AJ. Responses to amyloids of microbial and host origin are mediated through toll-like receptor 2. Cell Host Microbe. 2009;6(1):45–53. Epub 2009/07/21. doi: 10.1016/j.chom.2009.05.020 ; PubMed Central PMCID: PMC2745191. 19616765 PMC2745191

[20] Peng HL, Callender R. Mechanism for Fluorescence Quenching of Tryptophan by Oxamate and Pyruvate: Conjugation and Solvation-Induced Photoinduced Electron Transfer. J Phys Chem B. 2018;122(25):6483–90. Epub 2018/06/05. doi: 10.1021/acs.jpcb.8b02433 ; PubMed Central PMCID: PMC6038119. 29860828 PMC6038119

[21] Tian P, Boomsma W, Wang Y, Otzen DE, Jensen MH, Lindorff-Larsen K. Structure of a functional amyloid protein subunit computed using sequence variation. J Am Chem Soc. 2015;137(1):22–5. Epub 2014/11/22. doi: 10.1021/ja5093634 . 25415595

[22] Kalyoncu E, Ahan RE, Olmez TT, Safak Seker UO. Genetically encoded conductive protein nanofibers secreted by engineered cells. RSC Advances. 2017;7(52):32543–51. doi: 10.1039/C7RA06289C

[23] Deshmukh M, Evans ML, Chapman MR. Amyloid by Design: Intrinsic Regulation of Microbial Amyloid Assembly. J Mol Biol. 2018;430(20):3631–41. Epub 2018/07/19. doi: 10.1016/j.jmb.2018.07.007 ; PubMed Central PMCID: PMC6168361. 30017921 PMC6168361

[24] Van Gerven N, Klein RD, Hultgren SJ, Remaut H. Bacterial amyloid formation: structural insights into curli biogensis. Trends Microbiol. 2015;23(11):693–706. Epub 2015/10/07. doi: 10.1016/j.tim.2015.07.010 ; PubMed Central PMCID: PMC4636965. 26439293 PMC4636965

[25] Wong GCL. Electrostatics of rigid polyelectrolytes. Current Opinion in Colloid & Interface Science. 2007;11(6):310–5. Epub 16 Jan 2007. doi: 10.1016/j.cocis.2006.12.003

[26] Wong GC, Pollack L. Electrostatics of strongly charged biological polymers: ion-mediated interactions and self-organization in nucleic acids and proteins. Annu Rev Phys Chem. 2010;61:171–89. Epub 2010/01/09. doi: 10.1146/annurev.physchem.58.032806.104436 . 20055668

[27] Schmidt NW, Jin F, Lande R, Curk T, Xian W, Lee C, et al. Liquid-crystalline ordering of antimicrobial peptide-DNA complexes controls TLR9 activation. Nat Mater. 2015;14(7):696–700. doi: 10.1038/nmat4298 . 26053762

[28] Lee EY, Zhang C, Di Domizio J, Jin F, Connell W, Hung M, et al. Helical antimicrobial peptides assemble into protofibril scaffolds that present ordered dsDNA to TLR9. Nat Commun. 2019;10(1):1012. doi: 10.1038/s41467-019-08868-w ; PubMed Central PMCID: PMC6399285. 30833557 PMC6399285

[29] Rapsinski GJ, Wynosky-Dolfi MA, Oppong GO, Tursi SA, Wilson RP, Brodsky IE, et al. Toll-like receptor 2 and NLRP3 cooperate to recognize a functional bacterial amyloid, curli. Infect Immun. 2015;83(2):693–701. doi: 10.1128/IAI.02370-14 ; PubMed Central PMCID: PMC4294241. 25422268 PMC4294241

[30] Hathroubi S, Mekni MA, Domenico P, Nguyen D, Jacques M. Biofilms: Microbial Shelters Against Antibiotics. Microb Drug Resist. 2017;23(2):147–56. doi: 10.1089/mdr.2016.0087 . 27214143

[31] Hung C, Zhou Y, Pinkner JS, Dodson KW, Crowley JR, Heuser J, et al. Escherichia coli biofilms have an organized and complex extracellular matrix structure. mBio. 2013;4(5):e00645–13. doi: 10.1128/mBio.00645-13 ; PubMed Central PMCID: PMC3774191. 24023384 PMC3774191

[32] Zogaj X, Bokranz W, Nimtz M, Romling U. Production of cellulose and curli fimbriae by members of the family Enterobacteriaceae isolated from the human gastrointestinal tract. Infect Immun. 2003;71(7):4151–8. doi: 10.1128/IAI.71.7.4151-4158.2003 ; PubMed Central PMCID: PMC162016. 12819107 PMC162016

[33] McCrate OA, Zhou X, Reichhardt C, Cegelski L. Sum of the parts: composition and architecture of the bacterial extracellular matrix. J Mol Biol. 2013;425(22):4286–94. doi: 10.1016/j.jmb.2013.06.022 ; PubMed Central PMCID: PMC3812305. 23827139 PMC3812305

[34] Fowler DM, Koulov AV, Balch WE, Kelly JW. Functional amyloid—from bacteria to humans. Trends Biochem Sci. 2007;32(5):217–24. Epub 2007/04/07. S0968-0004(07)00059-X [pii] doi: 10.1016/j.tibs.2007.03.003 . 17412596

[35] Reed-Geaghan EG, Savage JC, Hise AG, Landreth GE. CD14 and toll-like receptors 2 and 4 are required for fibrillar A{beta}-stimulated microglial activation. J Neurosci. 2009;29(38):11982–92. Epub 2009/09/25. 29/38/11982 [pii] doi: 10.1523/JNEUROSCI.3158-09.2009 ; PubMed Central PMCID: PMC2778845. 19776284 PMC2778845

[36] Tahara K, Kim HD, Jin JJ, Maxwell JA, Li L, Fukuchi K. Role of toll-like receptor signalling in Abeta uptake and clearance. Brain. 2006;129(Pt 11):3006–19. doi: 10.1093/brain/awl249 ; PubMed Central PMCID: PMC2445613. 16984903 PMC2445613

[37] Halle A, Hornung V, Petzold GC, Stewart CR, Monks BG, Reinheckel T, et al. The NALP3 inflammasome is involved in the innate immune response to amyloid-beta. Nat Immunol. 2008;9(8):857–65. doi: 10.1038/ni.1636 ; PubMed Central PMCID: PMC3101478. 18604209 PMC3101478

[38] Hong W, Juneau RA, Pang B, Swords WE. Survival of bacterial biofilms within neutrophil extracellular traps promotes nontypeable Haemophilus influenzae persistence in the chinchilla model for otitis media. J Innate Immun. 2009;1(3):215–24. Epub 2009/01/01. doi: 10.1159/000205937 ; PubMed Central PMCID: PMC6951045. 20375579 PMC6951045

[39] Rada B. Interactions between Neutrophils and Pseudomonas aeruginosa in Cystic Fibrosis. Pathogens. 2017;6(1). Epub 2017/03/12. doi: 10.3390/pathogens6010010 ; PubMed Central PMCID: PMC5371898. 28282951 PMC5371898

[40] Holmqvist S, Chutna O, Bousset L, Aldrin-Kirk P, Li W, Bjorklund T, et al. Direct evidence of Parkinson pathology spread from the gastrointestinal tract to the brain in rats. Acta Neuropathol. 2014;128(6):805–20. doi: 10.1007/s00401-014-1343-6 . 25296989

[41] Sampson TR, Challis C, Jain N, Moiseyenko A, Ladinsky MS, Shastri GG, et al. A gut bacterial amyloid promotes alpha-synuclein aggregation and motor impairment in mice. Elife. 2020;9. doi: 10.7554/eLife.53111 ; PubMed Central PMCID: PMC7012599. 32043464 PMC7012599

[42] Ohnishi S, Koide A, Koide S. The roles of turn formation and cross-strand interactions in fibrillization of peptides derived from the OspA single-layer beta-sheet. Protein Sci. 2001;10(10):2083–92. Epub 2001/09/22. doi: 10.1110/ps.15901 ; PubMed Central PMCID: PMC2374230. 11567099 PMC2374230

[43] Alteri CJ, Xicohtencatl-Cortes J, Hess S, Caballero-Olin G, Giron JA, Friedman RL. Mycobacterium tuberculosis produces pili during human infection. Proc Natl Acad Sci U S A. 2007;104(12):5145–50. Epub 2007/03/16. doi: 10.1073/pnas.0602304104 [pii] ; PubMed Central PMCID: PMC1817835. 17360408 PMC1817835

[44] Besingi RN, Wenderska IB, Senadheera DB, Cvitkovitch DG, Long JR, Wen ZT, et al. Functional amyloids in Streptococcus mutans, their use as targets of biofilm inhibition and initial characterization of SMU_63c. Microbiology (Reading). 2017;163(4):488–501. Epub 2017/02/01. doi: 10.1099/mic.0.000443 ; PubMed Central PMCID: PMC5775903. 28141493 PMC5775903

[45] Chen X, Yang X, de Anda J, Huang J, Li D, Xu H, et al. Clostridioides difficile Toxin A Remodels Membranes and Mediates DNA Entry Into Cells to Activate Toll-Like Receptor 9 Signaling. Gastroenterology. 2020;159(6):2181–92 e1. Epub 2020/08/26. doi: 10.1053/j.gastro.2020.08.038 ; PubMed Central PMCID: PMC8720510. 32841647 PMC8720510

[46] Putsep K, Carlsson G, Boman HG, Andersson M. Deficiency of antibacterial peptides in patients with morbus Kostmann: an observation study. Lancet. 2002;360(9340):1144–9. doi: 10.1016/S0140-6736(02)11201-3 . 12387964

[47] Lande R, Botti E, Jandus C, Dojcinovic D, Fanelli G, Conrad C, et al. The antimicrobial peptide LL37 is a T-cell autoantigen in psoriasis. Nat Commun. 2014;5:5621. doi: 10.1038/ncomms6621 . 25470744

[48] Lande R, Chamilos G, Ganguly D, Demaria O, Frasca L, Durr S, et al. Cationic antimicrobial peptides in psoriatic skin cooperate to break innate tolerance to self-DNA. Eur J Immunol. 2015;45(1):203–13. doi: 10.1002/eji.201344277 . 25332209

[49] Lande R, Ganguly D, Facchinetti V, Frasca L, Conrad C, Gregorio J, et al. Neutrophils activate plasmacytoid dendritic cells by releasing self-DNA-peptide complexes in systemic lupus erythematosus. Sci Transl Med. 2011;3(73):73ra19. doi: 10.1126/scitranslmed.3001180 ; PubMed Central PMCID: PMC3399524. 21389263 PMC3399524

[50] Lande R, Gregorio J, Facchinetti V, Chatterjee B, Wang YH, Homey B, et al. Plasmacytoid dendritic cells sense self-DNA coupled with antimicrobial peptide. Nature. 2007;449(7162):564–9. doi: 10.1038/nature06116 . 17873860

[51] Pachucki R, Corradetti C., Kohler L., Ghadiali J., Gallo P., Nicastro L., Tursi S., Gallucci S., Tukel C., Caricchio R. Persistent Bacteriuria and Antibodies recognizing Curli/eDNA complexes from E. Coli are Linked to Flares in Systemic Lupus Erythematosus. Arthritis Rheum. 2020. 10.1002/art.41400 PMC7722165 32840064

[52] Nasution AR, Mardjuadi A, Kunmartini S, Suryadhana NG, Setyohadi B, Sudarsono D, et al. HLA-B27 subtypes positively and negatively associated with spondyloarthropathy. J Rheumatol. 1997;24(6):1111–4. . 9195518

[53] Larsen P, Nielsen JL, Dueholm MS, Wetzel R, Otzen D, Nielsen PH. Amyloid adhesins are abundant in natural biofilms. Environ Microbiol. 2007;9(12):3077–90. doi: 10.1111/j.1462-2920.2007.01418.x . 17991035

[54] Lim JY, May JM, Cegelski L. Dimethyl sulfoxide and ethanol elicit increased amyloid biogenesis and amyloid-integrated biofilm formation in Escherichia coli. Appl Environ Microbiol. 2012;78(9):3369–78. doi: 10.1128/AEM.07743-11 ; PubMed Central PMCID: PMC3346451. 22389366 PMC3346451

[55] Ilavsky J. Nika: software for two-dimensional data reduction. Journal of Applied Crystallography. 2012;(45):324–8. doi: 10.1107/S0021889812004037

[56] Hammersley A. FIT2D: an introduction and overview. European Synchrotron Radiation Facility Internal Report:ESRF97HA02T 1997.

[57] Tukel C, Raffatellu M, Humphries AD, Wilson RP, Andrews-Polymenis HL, Gull T, et al. CsgA is a pathogen-associated molecular pattern of Salmonella enterica serotype Typhimurium that is recognized by Toll-like receptor 2. Mol Microbiol. 2005;58(1):289–304. doi: 10.1111/j.1365-2958.2005.04825.x . 16164566

[58] Wilson RP, Raffatellu M, Chessa D, Winter SE, Tukel C, Baumler AJ. The Vi-capsule prevents Toll-like receptor 4 recognition of Salmonella. Cell Microbiol. 2008;10(4):876–90. doi: 10.1111/j.1462-5822.2007.01090.x . 18034866

[59] Sriram U, Varghese L, Bennett HL, Jog NR, Shivers DK, Ning Y, et al. Myeloid dendritic cells from B6.NZM Sle1/Sle2/Sle3 lupus-prone mice express an IFN signature that precedes disease onset. J Immunol. 2012;189(1):80–91. doi: 10.4049/jimmunol.1101686 ; PubMed Central PMCID: PMC3381850. 22661089 PMC3381850

[60] Noto Llana M, Sarnacki SH, Vazquez MV, Gartner AS, Giacomodonato MN, Cerquetti MC. Salmonella enterica induces joint inflammation and expression of interleukin-17 in draining lymph nodes early after onset of enterocolitis in mice. Infect Immun. 2012;80(6):2231–9. doi: 10.1128/IAI.00324-12 ; PubMed Central PMCID: PMC3370572. 22493084 PMC3370572

