# Supplementary figures and images for: Assembly of ordered DNA-curli fibril complexes during Salmonella biofilm formation correlates with strengths of the type I interferon and autoimmune responses
Source: PLoS Pathog. 2022 Aug 16;18(8):e1010742. doi: 10.1371/journal.ppat.1010742 (PMC9380926; doi:10.1371/journal.ppat.1010742)

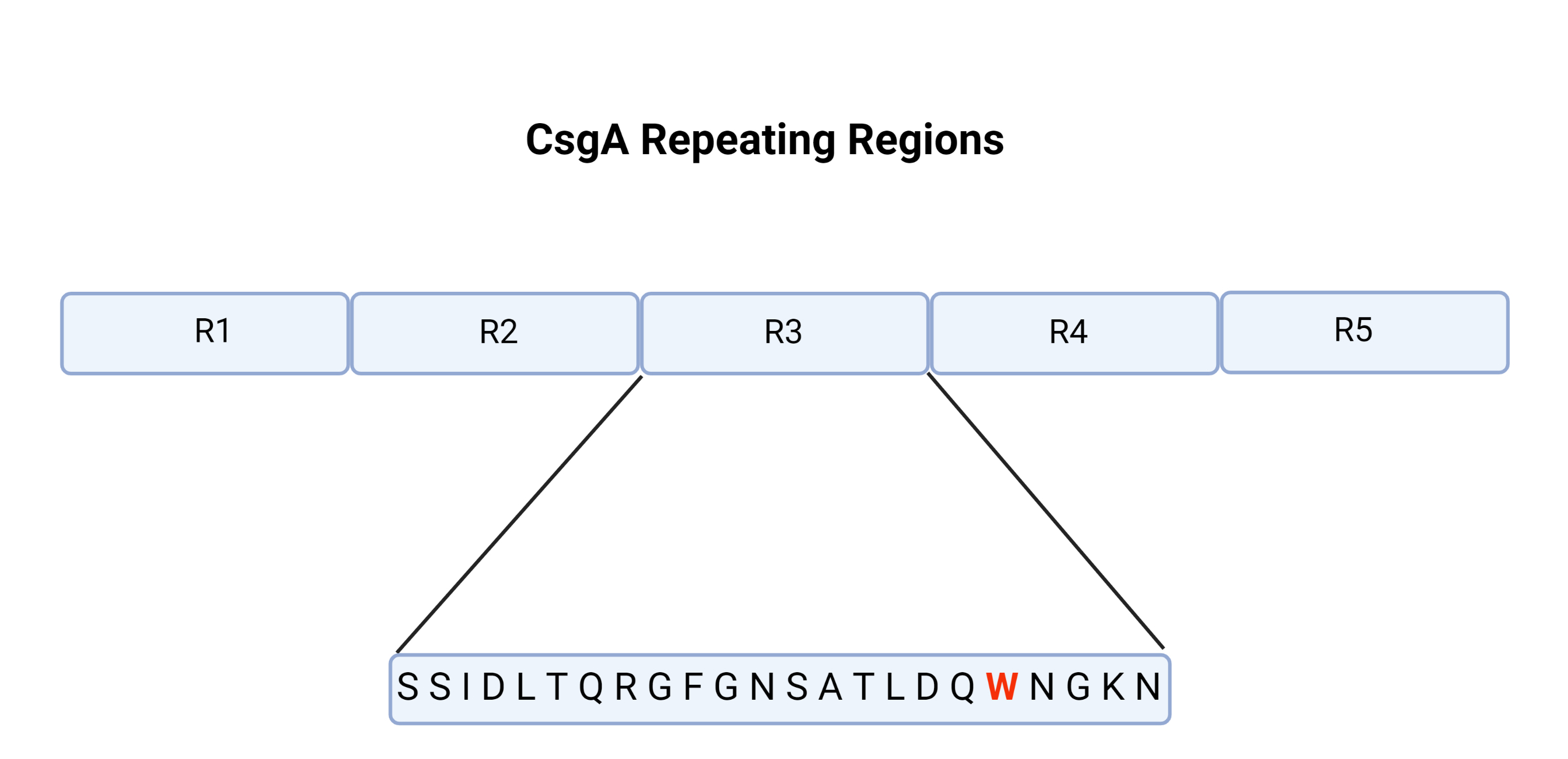

Supplement: S1 Fig — The amino acid structure of the CsgA monomer emphasizing the single tryptophan within the sequence used for the analysis of polymerization. (JPEG) [file ppat.1010742.s001.jpeg]
